# A Practical Guide for Intra-Renal Temperature and Pressure Management during Rirs: What Is the Evidence Telling Us

**DOI:** 10.3390/jcm11123429

**Published:** 2022-06-15

**Authors:** Felipe Pauchard, Eugenio Ventimiglia, Mariela Corrales, Olivier Traxer

**Affiliations:** 1 Urology Department, Hospital Naval Almirante Nef, Viña del Mar 2520000, Chile; felipepauchard@gmail.com; 2 Division of Experimental Oncology/Unit of Urology, URI, IRCCS Ospedale San Raffaele, 20132 Milan, Italy; eugenio.ventimiglia@gmail.com; 3 Groupe de Recherche Cliniques sur la Lithiase Urinaire, Hôpital Tenon, Sorbonne Université, F-75020 Paris, France; mariela_corrales_a@hotmail.com; 4 Service d’Urologie, Assitance-Publique Hôpitaux de Paris, Hôpital Tenon, Sorbonne Université, F-75020 Paris, France

**Keywords:** ureteroscopy, intrarenal temperature, intrarenal pressure

## Abstract

Introduction: One of the main limitations of Ho:YAG lithotripsy is represented by its advancement speed. The need for faster lithotripsy has led to the introduction of high-power laser equipment. This general trend in increasing Ho:YAG lithotripsy power has certain points that deserve to be considered and analyzed. The objective is to carry out a narrative review on intrarenal temperature and pressure during ureteroscopy. Methods: A literature search using PUBMED database from inception to December 2021 was performed. The analysis involved a narrative synthesis. Results: Using more power in the laser correlates with an increase in temperature that can be harmful to the kidney. This potential risk can be overcome by increasing either the irrigation inflow or outflow. Increasing irrigant flow can lead to high intrarenal temperature (IRP). The factors that allow the reduction of intrarenal pressure are a low irrigation flow, the use of a ureteral access sheath of adequate diameter according to the equipment used, and the occupation of the working channel by the laser or basket. Conclusion: To maintain a safe temperature profile, it has been proposed to use chilled irrigation fluid, intermittent laser activation or to increase irrigation flow. This last recommendation can lead to increased IRP, which can be overcome by using a UAS. Another option is to use low power laser configurations in order to avoid temperature increases and not require high irrigation flows.

## 1. Introduction

During the last 30 years, urology has undergone a very important development in minimally invasive surgery [1]. Holmium:YAG (Ho:YAG) laser lithotripsy has become the optimum standard for the treatment of stones with ureteroscopy [2]. One of the main limitations of Ho:YAG lithotripsy is represented by its advancement speed [3], which implies limiting the total operative time in the case of large stones in order to avoid treatment-related complications [4,5]. The need for faster lithotripsy has led to the introduction of high-power laser equipment (≥100 W) that allows for the reaching frequencies up to 120 Hz, as opposed to the 20 Hz of low-power laser generators (30 W), with the ultimate aim of reducing surgical times. This has led some authors to propose using extreme laser settings up to 2 J and 50 Hz during flexible uretoscopy [6].

This general trend in increasing Ho:YAG lithotripsy laser working power has certain points that deserve to be considered and analyzed. The objective of this manuscript is to carry out a narrative review to elucidate determinants of both intrarenal temperature (IRT) and pressure (IRP) during ureteroscopy, discussing possible issues arising when working with high-power settings.

## 2. Methods

A literature search using the PUBMED database from inception to December 2021 was performed. Keywords used were “intrarenal pressure”, “ureteroscopy” and “temperature”. Boolean operators (AND, OR) were used to refine the search. In vitro, human and animal studies were included.

Additional articles identified through references were also included. Original and review articles were included. The analysis involved a narrative synthesis.

### 2.1. Intra-Renal Temperature during Flexible Ureteroscopy

It is possible to estimate the efficiency of laser lithotripsy by using the proportion of emitted laser energy capable of reaching the stone. An in vitro study reported that, using a frequency of 20 Hz, only 52% of the emitted pulses reach the stone; when using frequencies of 50 Hz, only 23% reach the objective and only 4% at 80 Hz are reached [7]. Since it is virtually impossible to estimate this proportion in everyday life, a practical way to evaluate the efficiency of laser lithotripsy is represented by the ratio between total emitted energy and the treated stone volume, i.e., the joules/mm^3^ concept [8]. This ratio is supported by an in vitro study [9] that demonstrated how much energy is needed to ablate 1 mm^3^ of different stone compositions at perfect conditions. As a practical guide, it means that for a certain stone composition, the higher the ratio (Joules/mm^3^) needed to ablate the stone, the less efficient the lithotripsy was.

A comparative study between high and low power laser lithotripsy showed significantly less operative time but higher joules/mm^3^ values for the high power laser [10]. What happens with this extra delivered energy?

The use of high-power leads to greater energy delivery; not all the extra energy targets the stone and is absorbed by the water, increasing IRT. Also, these laser emissions that do not reach the stone can damage the urothelium and potentially produce complications (i.e., ureter strictures).

When does this become a concern?

The thermal damage of the tissues depends on the temperature reached and its maintenance over time (thermal dose), and this is how it has been considered that tissue and cell damage occur at 43 °C held for 240 min [11]. Thermal dose is a nonlinear function, so it takes roughly 15 s at 53 °C to produce damage [12]. Therefore, temperature control during ureteroscopy is of paramount importance.

In a laboratory study that simulated a renal calyx [13], the authors evaluated the working temperature during ureteroscopy. The study consisted of activating the laser with different configurations of energy and frequencies, measuring the temperature of the medium at different irrigation flows. It was seen that the higher the delivered energy, the greater the increase in fluid temperature, reaching temperatures of 50 °C and 70 °C at 10 and 60 s after laser activation without irrigation, respectively. This increase in temperature was lower when the irrigant flow increased. With an irrigant flow of 40 mL/min, the temperature did not exceed 38.5 °C after 10 and 60 s of laser activation, regardless of the laser configuration. The same study was subsequently carried out in pigs and similar results were obtained, reaching even higher temperatures as compared to the previously mentioned laboratory setting, which resulted in verified kidney damage (charred tissue at the urothelial surface in contact with the collecting system) after 60 s of laser activation. Therefore, the authors concluded that the use of high-power lasers with a 40 W configuration can induce harmful temperature rises in a porcine in vivo model [14]. Subsequently, a laboratory study evaluated different power thresholds of 5, 10, 20 and 40 W, concluding that when using power less than or equal to 20 W, safe temperatures were maintained while maintaining an irrigant flow of 15 mL/min. A higher irrigant flow, 40 mL/min, was needed with the laser firing at 40 W in order to avoid harmful rises in fluid temperature [12]. A similar study [15] simulating a stone treated in the ureter reported that using laser parameters of 10 W reach a safe temperature with 10 mL/min of irrigation. Another ex vivo study in pigs evaluated IRT with an irrigant pressure of 100 cm H_2_O, demonstrating that when using 40 W settings, irrigation flow should be increased, and a ureteral access sheath should ideally be used to counteract the rise in temperature [16].

The recent introduction of a novel super pulsed thulium fiber laser (TFL) looks very promising for endourologists [17]. How TFL changes the IRT during lithotripsy was evaluated in in vitro studies [18,19] which compare Ho:YAG with TFL, demonstrating no difference between lasers. An ex vivo study [20] in porcine kidneys evaluated temperature rise during ureteroscopy with manual pump irrigation of saline at room temperature comparing Ho:YAG with TFL at dusting (20–21 W) and fragmentation (6.4 W) settings. No rise of temperature above the threshold for potential cellular injury and no histological damage was seen with any of the lasers. Another study performed in pigs using TFL at different settings with irrigant pressure at 200 cm H_2_O showed IRT reaching or exceeding a 44 °C threshold. This was overcome using a 12/14 Fr ureteral access sheath (UAS) at room temperature irrigation and a laser set at 10 or 30 W. When 40 W was used, the temperature rose above the threshold despite UAS and high inflow [21].

In summary, using more power in the laser correlates with an increase in temperature that can be harmful to the kidney. This potential risk can be overcome by increasing either the irrigation inflow or outflow (i.e., by using UAS).

### 2.2. Intra-Renal Pressure during Flexible Ureteroscopy

The normal intrarenal pressure (IRP) range is 0–20 cm H_2_O [22]. An IRP between 27–41 cm H_2_O results in pyelotubular reflux; pressures between 41–68 cm H_2_O result in pyelovenous backflow and pressures of 81–95 cm H_2_O can result in fornix rupture. These increases in intrarenal pressure are related to infectious and hemorrhagic complications, as well as kidney damage [22,23,24]. When working with an irrigation flow greater than 6 mL/min, the ureter behaves like an open tube, resulting in a linear relationship between flow and pressure [23], so caution must always be exercised when working in the ureter.

It is important to remember that to achieve an irrigation flow of 7–8 mL/min occupying the 3.6 Fr working channel of a flexible ureteroscope with a 200 µm laser fiber, the irrigation bag must be hung at 60 cm H_2_O over the tip of the ureteroscope to achieve a flow of 14–15 mL/min at 100 cm H_2_O and for 40 mL/min at 304 cm H_2_O [13].

The pressure achieved during a ureteroscopy varies by many factors such as irrigation flow, the use of a ureteral access sheath, and a free or occupied working channel. Thus, during a ureteroscopy without a UAS using a Storz Flex X2 7.6 Fr ureteroscope (Karl Storz, Tuttlingen, Germany) with an irrigation pump at 8 mL/min with hand held irrigation support with a 20 mL syringe, intrarenal pressures up to 328 mmHg can be reached [22].

Recently, the kidney damage caused by high intrarenal pressure was studied in pigs using an Olympus P5 8.4 Fr (Olympus, Center Valley, PA, USA) with a free working channel, at different irrigation pressures (68–272 cm H_2_O) with and without ureteral access sheaths 12/14 Fr (Flexor Cook Medical, Bloomingotn IN, USA). The authors observed pressures ranging from 40–167 cm H_2_O when not using a ureteral access sheaths and significantly lower pressures when using a sheath, which was correlated with increased penetration of irrigant and parenchymal damage [25].

Using a LithoVue f-URS 9,5 Fr (Boston Scientific, Marlborough, MA, USA) with a 12/14 Fr ureteral access sheath has been shown in laboratory studies to significantly decrease IRP when compared to the use of smaller diameter sheaths or even without the use of a sheath, maintaining an irrigation pressure of 40 cm H_2_O [26]. Occupying the working channel of the ureteroscope with a 273 µm fiber laser significantly reduces the intrarenal pressure, except in cases where a 10/12 Fr ureteral access sheath is used with an irrigation pressure of 193 cm H_2_O.

An ex vivo study [27] in porcine kidneys evaluated intrarenal pressure using a Storz Flex X2 7.6 Fr ureteroscope (Karl Storz, Tuttlingen, Germany) with a 200 µm laser fiber. The authors demonstrated that hanging the irrigation bag at 100 cm H_2_O above the patient gives a 10–15 mL/min irrigation flow, and the intrarenal pressure reached 33–38 and 14–15 cm H_2_O with 10/12 and 12/14 Fr UAS, respectively.

It should be taken into consideration that, despite demonstrating a decrease in IRP, the use of a ureteral access sheath seems to be decreasing with a reported use of only 21% of cases in a series that describes the experience with laser configurations of high power [28].

In an in vivo study with and without the use of a 12/14 Fr ureteral access sheath, the authors measured IRP during ureteroscopy using a nephrostomy tube, identifying pressures over 55 cm H_2_O with the sheath and 128 cm H_2_O without the sheath [29]. Another recently published experience studied IRP in vivo using a sensor wire during flexible ureteroscopy, using or not using a ureteral access sheath with constant irrigation pressure of 80 cm H_2_O and with manual pump support, demonstrating intrarenal pressures over 100 cm H_2_O in all evaluated scenarios and reaching maximum pressures over 300 cm H_2_O, regardless of whether a 10/12 Fr, 12/14 Fr UAS was used or without the use of a sheath [30]. These studies show that the use of a UAS alone does not ensure safe intrarenal pressure. It should also be noted that the real impact on renal parenchyma of working at high intrarenal pressure constantly or intermittently (i.e., manual pump) has not been studied and needs to be evaluated.

There are plenty of ureteroscopes in the market with many different diameters. If UAS is to be used, we should aim to choose the best compromise between the diameter of the scope and the UAS. An in vitro study [31] evaluated the intrarenal pressure combining the most common reusable ureteroscopes and different UAS diameters at a constant irrigation pressure of 60 cmH20 (7–8 mL/min irrigation flow) and with a 272 µm fiber laser or empty working channel. The authors demonstrated that an occupied working channel at that irrigation pressure achieves safe intrarenal pressure at all different scenarios, and they conclude that a 10/12 UAS showed a good compromise between irrigation flow and IRP.

In summary, the factors that allow the reduction of intrarenal pressure are a low irrigation flow, the use of a ureteral access sheath of adequate diameter according to the equipment used, and the occupation of the working channel by the laser or basket [32].

### 2.3. Managing Intra-Renal Temperature and Pressures: Tips and Tricks

Considering the increases in intrarenal pressure that can be obtained by working at high irrigation flow to decrease the temperature using high power in the laser, it has been proposed to perform ureteroscopy with cooled irrigant solution, showing in an in vitro study that the temperature reached in the renal cavities would not be harmful when activating the laser at a 40 W setting and maintaining an irrigation flow of 12 mL/min of fluid at 1 °C [33]. This seems to be a valid alternative; however, we do not know if performing a ureteroscopy with an irrigation solution cooled to 1 °C at medium flow is safe or not for the patient.

Another option that has been proposed to reduce temperature rises during the use of high-power settings is the intermittent activation of the laser for 3–4 s and pausing for another 3–4 s [34]. Aldhouki et al. [35] showed that after 9 s of continuous firing at 40 W with irrigant at 8 mL/min, thermal damage may occur, but when using the same power with a 5 s interval with the pedal on/off no thermal injury occurred. Furthermore, no thermal injury occurred when using 20 W with continuous firing. There are two possible ways to push the pedal: discontinuously or continuously. Which technique to use depends on what laser parameters we are using. However, the discontinuous technique seems to make no sense if what we are looking for when using high frequencies is to reduce operative times.

A third recommendation to avoid high temperatures is to increase irrigation flows [34], but as previously mentioned, this can lead to increased intrarenal pressure and eventual complications. UAS can reduce intrarenal pressure, but which UAS to use depends on several factors. The first involves the ureteral anatomy. Second, the diameter of the ureteroscope that we are using is a factor; and, finally, it depends on how much flow we want to irrigate. Combining these three factors, the power of the laser can be adjusted to achieve safe temperature and pressure parameters.

It should be noted that stone fragments/dust can partially obstruct irrigation outflow through the ureter during stone treatment and eventually increase intrarenal pressure up to the irrigation pressure that is being used, so it seems that it might be safer to use low levels of irrigation.

Finally, another option is to use low power laser configurations to avoid temperature increases and not require high irrigation flows, as was shown by Teng et al. [36], who measured in vivo IRP and IRT using low power (<20 W) and moderate irrigation flow (15–30 mL/min) while maintaining safe temperature profiles. Table 1 and Table 2 summarize risks, determinants and possible solutions for IRP and IRT.

Considering that a recent meta-analysis did not demonstrate superiority in terms of a shorter surgical time with high-power lasers compared to low-power lasers when the same stone volumes were assessed [3], it seems reasonable to consider the previous points and assess whether the potential risks to which patients are being exposed are justified with regard to using this type of laser setup today.

Most of the literature reviewed are based on in vitro studies, and we know that real life does not always behave as things do in the laboratory. More studies are needed to allow us to better understand the real risks and limitations that we should have at the time of performing a ureteroscopy. Thus, the recommendations stated in Table 3 must be taken with caution.

Furthermore, having equipment that allows for the measuring of temperature and intrarenal pressure continuously during surgery would allow for better control of these variables, since ureteroscopes currently lack this technology, and the only way to measure them is with invasive methods (renal puncture or sensor wire).

## 3. Conclusions

This narrative review found that laser power, IRP and IRT are strictly related and must be considered during intrarenal surgery to avoid potential kidney damage. Most of the studies referenced were not in humans and recommendations must be taken with caution.

## Figures and Tables

**Table 1:** Summary of risks and potential solutions.

Risks	Solutions
High temperature: - Renal damage	Increase irrigation flow Decrease laser power Pauses during laser activation Chilled irrigation fluid?
High pressure: - Renal damage - Infection - Bleeding	Decrease irrigation flow Use of Ureteral access sheath Occupy working channel

**Table 2:** Determinants of high temperature and pressure.

High Temperature	High Pressure
Decrease irrigation flow Increase laser power	Increase irrigation flow No use of Ureteral access sheath Empty working channel

**Table 3:** Safe temperature settings during ureteroscopy.

	Safe Temperature Setting	
Laser power	Irrigation flow	Saline bag height
10 W	10 mL/min	>60 cm H_2_O *
20 W	15 mL/min	100 cm H_2_O
40 W	40 mL/min	304 cm H_2_O

* Saline bag hung at 60 cm above the patient achieve 7–8 mL/min with the working channel occupied with a 200 micron laser fiber. In order to achieve 10 mL/min it should be hung higher than 60 cm and below 100 cm. We did not find a study that evaluated the height needed to achieve that flow.

## Data Availability

Not applicable.
